# Focusing Attention to Improve Throwing Skills in Children With Autism Spectrum Disorder: Exploring the Influence of Working Memory

**DOI:** 10.1155/oti/8855971

**Published:** 2025-01-03

**Authors:** Qaith Mohammad Ramadan, Ayoob Sabaghi, Ali Heirani

**Affiliations:** Department of Motor Behavior, Faculty of Physical Education and Sport Sciences, Razi University, Kermanshah, Iran

**Keywords:** attention, autism, children, external, internal, working memory

## Abstract

This study is aimed at investigating the impact of internal and external attention focus on learning a throwing skill in children with autism, as well as the relationship between working memory and learning rate. Twenty-four children aged 6–8 years with autism were assigned to internal and external attention groups. Participants performed a throwing task while their working memory was assessed using Cornoldi's working memory test. The data was analyzed using ANOVA with repeated measures involving two attention instructions and five blocks during the acquisition stage. An independent *t*-test was conducted during the retention phase. Furthermore, a Pearson correlation test was utilized to explore any potential relationship between working memory and performance in both the acquisition and retention stages. Data analysis revealed no significant difference between the internal and external attention groups during the acquisition phase (*p* > 0.05), but a significant difference was found in the retention phase (*p* < 0.05). There was no correlation between working memory and learning outcomes (*p* > 0.05). The results suggest that internal attention may enhance motor learning in children with autism, and reducing working memory load does not necessarily favor external attention.

## 1. Introduction

Autism spectrum disorder (ASD), as outlined in the statistical and diagnostic manual of mental disorders, presents a significant challenge. This condition is characterized by difficulties in communication and social interaction (SI), along with repetitive behaviors and restricted interests. Symptoms of ASD can vary in severity, with some individuals exhibiting mild manifestations while others experience more pronounced symptoms [1]. Research indicates that individuals with ASD often encounter challenges in executing movements proficiently, with a recent review suggesting that a substantial percentage of autistic children—ranging from 50% to 88%—may exhibit notable motor impairments [2–4]. Learning and performing motor skills can be particularly challenging for individuals with ASD, highlighting the importance of developing effective strategies to enhance their motor skill acquisition.

One method to enhance learning involves providing attention instructions [5]. These instructions are categorized into internal focus and external focus. Internal focus involves directing attention towards body movements, while external focus involves focusing on the impact of movements on the environment [6]. Research consistently demonstrates that adopting an external focus of attention leads to improved motor performance and learning compared to an internal focus [7–9] This advantage is attributed to the constrained action hypothesis [10], which suggests that an external focus promotes automatic and reflexive motor control, resulting in more effective movement outcomes [11, 12]. It has been proposed that individual differences may influence the effects of attentional focus instructions [5].

However, only a small number of studies have investigated the impact of different attention foci on learning in children with atypical development, including those with attention deficit hyperactivity disorder, intellectual disability, or ASD [13–17]. In a study focusing on children with autism, Tse observed that internal attention facilitated enhanced learning outcomes during a throwing task in children aged 9–12 [15]. Similarly, Khodadadeh, Sabaghi, and Ebrahimi reported that internal attention was associated with superior learning outcomes relative to external attention during a retention test (mean age = 6.94 ± 0.42) [17]. In contrast, Asadi et al. found that, among children with autism engaged in observational learning, external attention resulted in better learning outcomes compared to internal attention [16].

Previous research has suggested that working memory capacity may influence how individuals respond to different types of attentional focus instructions [18] and working memory is not restricted to cognitive tasks; practice and learning of motor skills can also require the involvement of working memory. This includes the conscious correction of movement errors to develop execution strategies [18]. It has been reported that working memory is essential for holding relevant information (such as previous errors, the order of movements, or instructions) while performing a skill. Evidence suggests that working memory plays a role in executing movements. For example, when multiple instructions are provided, the ability to enact them is positively associated with working memory capacity [19, 20]. It is posited that environments that place high demands on working memory facilitate better learning for individuals with higher working memory capacity [21, 22]. While some studies have examined the general relationship between working memory and motor learning, there remains a gap in understanding how working memory correlates with performance under different attentional focus conditions, particularly in children with autism. Thus, this study is aimed at comparing the effects of internal and external attention on motor skill learning in children with autism and at investigating the connection between learning and working memory within this demographic. Despite the recognized difficulties with working memory in individuals with autism [23, 24], it remains to be seen whether they can benefit from internal attention strategies, which typically demand greater working memory resources.

## 2. Method

This quasiexperimental study was designed with a pretest, acquisition phase, and retention test. The statistical population consisted of boys aged 6–8 with ASD in Kermanshah Province. The sample size was calculated using G⁣^∗^Power 3.1.9.2 software. For this calculation, an effect size of *d* = 0.30, a statistical power of 90%, and the ANOVA test with repeated measures were considered. The results indicated that a minimum of 20 participants were required to meet these statistical conditions. However, due to increased participant interest, 12 individuals were included in each group.

Participants had to meet specific criteria, including a diagnosis of autism, IQ above 80, being boys aged 6–8, and being inexperienced in throwing skills. Exclusions were made for individuals with sensory or motor impairments, certain medications, or prior similar interventions. Medical records confirmed the absence of hearing or vision impairments and the nonexistence of attention deficit hyperactivity disorder. Participants' motor skills were evaluated through tasks involving catching and throwing a ball to assess balance and vision capabilities. Their auditory abilities were assessed through instructions related to ball tossing speeds. Primary hand preference was determined by observing which hand participants used to throw the ball. Parental consent was obtained for each participant.

### 2.1. Procedure

Based on the results of the pretest, in which participants completed a task (one block of 10 trails) of throwing beanbags towards a target placed on the ground, they were divided into two homogeneous groups: One focused on internal attention and the other on external attention. The internal attention group received instructions focusing on bodily movements, whereas the external attention group's instructions directed their focus away from their own bodies [15]. For a comprehensive list of the feedback expressions utilized in the internal and external attention groups, please consult Table 1.

The beanbag experiment was divided into three phases: pretest, acquisition, and retention test. Participants were directed to throw a beanbag at a target board, aiming for the center. Prior to the acquisition phase, participants received instructions on how to hold the beanbag and their stance, as well as a demonstration of the throwing motion. They were then assigned either internal or external attentional focus instructions, which were reiterated before each set of trials. Following the pretest (one block of 10 trials), the acquisition phase began 24 h later. To prevent fatigue from affecting the results of the acquisition test, there was a 24-h delay between the pretest and acquisition phase. During the acquisition phase, participants completed a total of 45 trials (five blocks of nine trials) within a single time period, with a 5-min rest period between blocks. The retention test took place 24 h after the acquisition phase (one block of 10 trials). Before the beginning of the acquisition phase, the subjects' working memory was measured with Cornoldi's working memory test (CWMT).

## 3. Measurement Tool Classification

### 3.1. Basic Assessment Tools

#### 3.1.1. Gilliam Autism Rating Scale 3 (GARS-3)

The GARS-3 has 58 items measuring specific ASD symptoms and associated difficulties in six subscales: restricted/repetitive behaviors (RRB; 13 items), SI (14 items), social communication (SC; nine items), emotional responses (ERs; eight items), cognitive style (CS; seven items), and maladaptive speech (MS; seven items). Each item has the same response format ranging from 0 (*not at all like the individual*) to 3 (*very much like the individual*). Within each subscale, a raw score is created as a sum of all answered items, where higher scores indicate more ASD symptoms/difficulties present. For nonverbal children, raw scores are derived from 44 items from the RRB, SI, SC, and ER subscale, while for verbal children, from all 58 items and the six subscales. Thus, there are four raw scores for an individual who is nonverbal (RRB, SI, SC, and ER) and six for a verbal individual (RRB, SI, SC, ER, CS, and MS). Using percentile ranks and norms, scaled scores are derived based on each domain's sum of scores, which is used to generate an autism index. An autism index of 54 and below indicates less likelihood of having ASD, while an autism index between 55 and 70 indicates the probability of ASD and Level 1 of ASD, which is based on DSM-5 classification and requires minimal support. An autism index score between 71 and 100 (Level 2 and requiring substantial support) and over 101 (Level 3 and requiring very significant support) indicates the very likely presence of ASD [25].

#### 3.1.2. Raven's Intelligence Test (Children's Version)

Raven's progressive matrices are a nonverbal test, which was established in 1938 by Raven and was revised in 1956 and is composed of 36 matrices or designs which in each, a part has been removed. The subject should find a removed part from six different options. The test has been prepared and published for children 5–11 years old and adults with mental retardation. And now, it is a common test for measuring the IQ in all counseling centers. Reliability and validity of Raven's progressive matrices have been confirmed in previous studies (the Cronbach alpha coefficient for internal consistency was 0.95 and for reliability *r* = 0.961) [26].

### 3.2. Working Memory Assessment Tool

#### 3.2.1. Active Memory Matrix Test

##### 3.2.1.1. CWMT

CWMT, also known as the working memory or active matrix, was designed by Cornoldi. The test consists of a 3-by-3 matrix with only one moving black square at the bottom left and the black square as the starting point of the test. In this test, the examiner asks the subject to look closely at the matrix and the square of the black square; then listen carefully to instructions that include moving up, down, left, or right of the black square and through mental imagination; and show the new place of the black square as directed. The score of each subject is calculated based on success in each stage. For each successful step of each command, one point is given, and in case of failure, zero point is given. In total, the subject will get a score between 0 and 3. The reliability of this test was reported favorably in many studies [27–29].

### 3.3. Throwing Skill Evaluation

#### 3.3.1. Apparatus and Task

**Figure 1:**
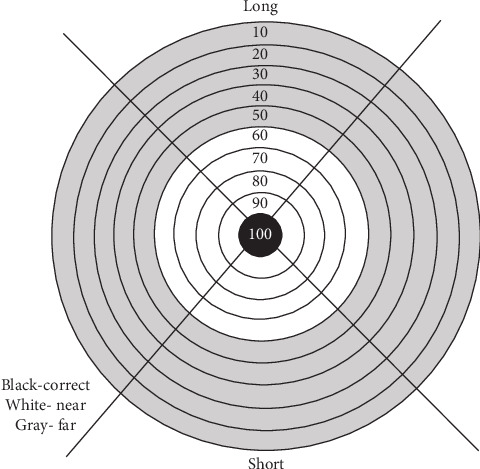
Schematic of the target and zone areas used for providing feedback [30].

The apparatus, task, and procedure were consistent with those used in previous studies (Figure 1) [30–32], and it has also been used in children with autism [30]. In this task, participants used their nondominant arm to toss beanbags towards a circular target on the floor. The target had a radius of 10 cm and was positioned 3 m away from the participants. Concentric circles with increasing radii of 20, 30, 40, 50, 60, 70, 80, 90, and 100 cm surrounded the target, creating zones to evaluate the accuracy of each throw. If the beanbag landed on the target itself, participants received 100 points. Throws landing in other zones earned 90, 80, 70, 60, 50, 40, 30, 20, or 10 points, depending on the proximity to the center, while those landing outside the circles scored zero. If a beanbag landed on a line between two zones, the participant was awarded the higher of the two scores.

### 3.4. Statistical Analysis

The statistical package SPSS Version 25.0 was used to analyze the data. To evaluate the differences between the two study groups in outcomes, in the acquisition phase, ANOVA test with repeated measures 2 (attention instructions) × 5 (Blocks 1–5) was used. Also, independent *t*-test was used in the retention phase. Also, the Pearson correlation test was used to check the relationship between working memory and acquisition and retention stages. The Shapiro–Wilk test was used to determine the assumption that the data were normal. Results were considered statistically significant when *p* values were < 0.05. Data is presented as mean ± standard deviation (SD).

## 4. Results

In this research, 24 children with ASD participated. In Table 2, the average and SD of their scores obtained of the subjects are reported.

**Figure 2:**
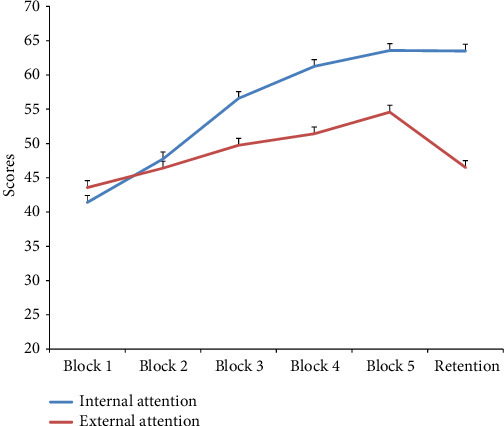
Mean ± SD of the average result of throwing task in different stages of learning.

As shown in Table 2, the groups were homogeneous and there was no difference between the studied groups before applying the intervention (*p* > 0.05), and Figure 2 shows the scores of the acquisition and retention stages of the throwing test.

As can be seen in Figure 2, the average scores increase during the acquisition phase (Blocks 1–5). The results of ANOVA analysis with repeated measurements showed that there is a significant difference between the experimental blocks (*F*_2.43,53.62_ = 20.246, *p* < 0.001, *ƞ*_*p*_^2^ = 0/47). However, the interaction of blocks in the group (*F*_2.44, 53.81_ = 2.957, *p* = 0.051, *ƞ*_*p*_^2^ = 0/11) was not significant and the group effect (*F*_1, 22_ = 0.651, *p* = 0.428, *ƞ*_*p*_^2^ = 0/02) did not reach a significant level in the acquisition phase. Unlike the acquisition stage, the group effect was observed in the retention stage (*p* < 0.05) and the independent *t*-test results showed that internal attention leads to more learning compared to external attention (*t*_22_ = 2.23, *p* = 0.036).

The results of the Spearman correlation analysis, presented in Table 3, examine the relationship between working memory and performance on the throwing task across each block in the acquisition phase. The data indicate a positive relationship between working memory and task performance in the blocks; however, none of these correlations reached statistical significance (*p* > 0.05). Additionally, during the retention phase, no significant positive correlation was found between working memory and the results of the retention test (*p* > 0.05).

## 5. Discussion

This study is aimed at examining how external and internal attention instructions impact the learning of a throwing skill in children with autism aged 6–8 years, focusing on working memory. Results indicated that during the acquisition phase, there was no significant difference between external and internal attention instructions, although the internal focus group showed better performance. However, in the retention phase, the internal focus group demonstrated significantly superior results compared to the external focus group. Additionally, there was no significant positive relationship between working memory and throwing task performance in either the acquisition or retention phase of learning.

Our results showed that using internal attention instructions led to better learning performance during the acquisition phase for children with autism, outperforming the results seen with external attention instructions. This finding contrasts with previous research on typically developing children [8, 9]. However, these findings align with the results reported by Tse and Khodadadeh, Sabaghi, and Ebrahimi, who demonstrated the advantages of employing an internal focus of attention over an external focus during the skill acquisition phase. Specifically, their studies highlighted a reduction in errors among participants utilizing an internal focus of attention compared to those directed towards an external focus [15, 17]. These results suggest that directing learners' focus towards the outcomes of their actions may not be effective for all individuals or situations. In the retention test, it was observed that internal attention instructions led to superior learning outcomes compared to external attention directives. This contrasts with the findings of Asadi et al., who found external attention to be more effective following video modeling. The discrepancy between the two studies could be attributed to differences in methodology [16]. Conversely, these findings are consistent with the research conducted by Tse and Khodadadeh et al., both of which underscored the advantages of internal attention [15, 17].

One possible explanation for these findings may be the heightened proprioceptive perception in children with autism. Proprioception involves the awareness of body posture, movement, and the relative positions of body parts [33, 34]. Interestingly, studies have indicated that children with autism tend to rely more on proprioception than vision when learning motor skills. For instance, research on the “rubber hand illusion” has shown that children with ASD are less susceptible to the illusion compared to typically developing children. Similarly, children with autism have been found to perform better on tasks that require attention to inner physiological processes, such as heartbeat counting [35–38]

Furthermore, studies have demonstrated that children with ASDs excel in tasks guided by proprioceptive information but struggle when visual errors are involved. This suggests that children with ASD may exhibit a reduced endpoint bias and rely more heavily on proprioceptive cues compared to typically developing children This stronger dependence on proprioception in individuals with ASD could potentially influence their ability to perceive conflicts between sensory inputs [33, 39].

Another reason for the superior performance of the internal attention group in younger children could be their inclination towards utilizing the motor system as a feedback source to enhance motor behavior and awareness. By focusing on their own body movements, children may experience improved kinetic abilities, leading to better motor performance [40]. Conversely, when children are directed to pay attention to the outcomes of their movements (external attention instructions), they heavily rely on their visual system for processing information. Research has shown abnormalities in integrated processing tasks that manipulate visual attention in individuals with autism [41]. Additionally, a study suggests that children with autism may exhibit an imbalanced sense of visual focus, often engaging in eye manipulation or stimulation without a clear external stimulus, as if they are searching or fixating on something in their environment [42]. These findings imply that children with autism may not fully benefit from external attention focus that depends on the visual system for information processing.

In addition to the reliance on proprioceptive perception and challenges with visual processing seen in individuals with autism, children tend to gravitate towards focusing on their own bodies when learning motor skills. A study conducted by Emanuel, Jarus, and Bart revealed that even children in the external attention group paid close attention to their hand movements while throwing darts [40]. Similarly, research by Tse on children with autism indicated that many participants fixated on their hand movements regardless of their assigned attention group. Intriguingly, those in the internal attention group maintained their focus on their own body movements throughout the dart-throwing task [15]. These findings suggest a preference among young children and individuals with autism for internal attention when acquiring motor skills.

The absence of a correlation between working memory and performance in the acquisition and retention phases aligns with previous studies by van Abswoude et al. and Brocken, Kal, and van der Kamp [5, 43] but contrasts with the findings of Buszard et al. [44]. The discrepancy with Buszard et al. may be attributed to the more complex nature of the basketball shooting task used in their study compared to the task in our research. In relation to the lack of association between working memory and outcomes in our study, it is important to consider that various studies have consistently shown the effectiveness of external attention over internal attention during these phases. These findings are often supported by the constrained action hypothesis proposed by Wulf, McNevin, and Shea, which suggests that external attention reduces the cognitive load on working memory, leading to enhanced performance, while internal attention may overload working memory and result in poorer performance for individuals focusing internally [10]. In our current study, we found different results compared to the constrained action hypothesis proposed by Wulf, McNevin, and Shea. Surprisingly, the group that focused on internal attention actually demonstrated superior performance. This unexpected outcome may be due to the developmental stage of the participants, who were children in our study. Children may not utilize working memory as efficiently as adults, as working memory performance typically improves from childhood to adolescence, with adults generally having larger memory [45].

Additionally, there is a developmental aspect to working memory performance, with task performance typically improving from childhood to adolescence. In contrast, adults typically have greater memory capacities than children [46]. Considering these developmental differences, it is possible that the benefits of external attentional feedback during motor skill acquisition are overshadowed by the cognitive strategies employed by young learners. Young learners often prioritize encoding and decoding new information, and their limited ability to use advanced memory strategies may negatively impact their motor performance [47, 48]. Moreover, when examining children with autism, their cognitive approaches and the processing of information seem to diverge from those of typically developing children. Studies indicate that individuals with autism frequently display deficiencies in both verbal and visual–spatial working memory [49]. Furthermore, it has been observed that individuals with autism often struggle with visual–spatial working memory. These individuals may face challenges in retaining, sustaining, and recalling visual–spatial information [50]. The deficits in working memory among individuals with autism can affect various cognitive domains such as adaptability, focus, and sustained attention [51]. While they may comprehend and retain the information, their ability to manipulate and use that information may be disrupted [52, 53].

### 5.1. Limitations

This study has several limitations that should be considered when interpreting the results. First, the small sample size (24 children) may limit the generalizability of the findings to the broader population of children with autism. Additionally, the age range of 6–8 years may not capture developmental differences in motor learning and working memory across different age groups, which limits the applicability of the results to older children or adolescents. The simplicity of the throwing task used may not have sufficiently challenged the participants' working memory or attention, suggesting that more complex tasks might yield different results. Finally, while the study hypothesized that children with autism rely more on proprioception than visual processing, the mechanisms behind this remain unclear, and further research is needed to explore how these sensory modalities interact during motor learning in children with autism. These limitations highlight areas for future research to provide a more comprehensive understanding of attention, working memory, and motor skill acquisition in this population.

## 6. Conclusion

In our recent study, we observed that children with autism, aged 6–8, showed improved motor learning in a throwing task when given internal attention instructions compared to external attention instructions. This preference for internal attention in this group may be due to their reliance on proprioceptive senses for motor skill learning and challenges in processing visual cognitive information emphasized in external attention instructions. Interestingly, we did not find a significant relationship between working memory and the learning stages, possibly due to underdeveloped memory system strategies in younger children and common working memory optimization issues in children with autism. Additionally, the simplicity of the task may have influenced this lack of relationship. Therefore, we recommend further research using more complex tasks for a more comprehensive understanding. In conclusion, our findings suggest that internal attention leads to enhanced learning in a throwing task for younger children with autism compared to external attention.

## Figures and Tables

**Table 1:** Attentional focused instruction [15].

**Internal focus**	**External focus**
Before throwing, concentrate on your arm position. Also, pay attention to your elbow movement	Look at the target attentively for a few seconds
Bring your hand backward until the beanbag touches your ear. At the end of the throw, your elbow is fully straightened	While throwing the beanbag, concentrate on its flight directly towards the target

**Table 2:** Descriptive statistics and independent *t*-test results for age, GARS-3 scores, Raven's test, and Cornoldi's test variables.

**Factors**	**Internal attention**	**External attention**	**t**	**df**	**p**
Age	6.95 ± 0.59	7.02 ± 0.41	0.15	22	0.88
GARS-3	70.83 ± 12.37	72.75 ± 10.02	1.02	22	0.31
Raven's test	87.41 ± 8.10	90.08 ± 9.44	0.74	22	0.46
Cornoldi's test	1.08 ± 0.66	1.25 ± 0.86	0.52	22	0.60

**Table 3:** Correlation between working memory and block scores during the acquisition and retention phases.

**Factors**	**Groups**	**Block 1**	**Block 2**	**Block 3**	**Block 4**	**Block 5**	**Retention phase**
Scores of the throwing test	Internal attention	41.41 ± 7.31	47.75 ± 7.31	56.58 ± 15.38	61.25 ± 14.13	63.58 ± 14.94	63.50 ± 16.54
	External attention	43.58 ± 16.38	46.41 ± 7.31	49.75 ± 18.62	51.41 ± 19.90	54.58 ± 23.56	46.50 ± 20.52

Scores of the working memory	Internal attention	1.08 ± 0.66	1.08 ± 17.71	1.08 ± 0.66	1.08 ± 0.66	1.08 ± 0.66	1.08 ± 0.66
	External attention	1.25 ± 0.88	1.25 ± 0.88	1.25 ± 0.88	1.25 ± 0.88	1.25 ± 0.88	1.25 ± 0.88

Spearman correlation	*r*	0.033	0.180	0.304	0.162	0.257	0.078
	*p*	0.879	0.399	0.149	0.449	0.226	0.718
	*N*	24	24	24	24	24	24

## Data Availability

The data that support the findings of this study are available from the corresponding author upon reasonable request.
